# Gut–Brain Axis and Mood Disorder

**DOI:** 10.3389/fpsyt.2018.00223

**Published:** 2018-05-29

**Authors:** Lu Liu, Gang Zhu

**Affiliations:** Department of Psychiatry, The First Affiliated Hospital of China Medical University, Shenyang, China

**Keywords:** intestinal microbiota, gut–brain axis, neuro-endocrine-immune, mood disorders, stress

## Abstract

Humans have over 100 trillion bacteria, highly abundant in the intestinal tract. Evidence suggests that intestinal microbiota is associated with the neuro-endocrine-immune pathways and can be associated with various mood disorders. This review summarizes findings from studies looking into neurobiochemical, neuroendocrine, and neuroimmune system mechanisms of the gut–brain axis to determine the relationship between intestinal microbiota and mood disorders. The effect of prebiotics, probiotics and antibiotics on mood disorders are also discussed, with the aim to propose some new therapeutic strategies for mood disorders.

## Introduction

The human body is composed of a complex biological system; with over 90% of microbiota cells and 10 million microbiota genes (1), it possesses over 100 trillion bacteria, 10 times the number of human body cells, 150 times the number of human genes, 1,000 species and over 7,000 strains (2). The intestinal tract is the most abundant area with bacterial concentration ranging from 10 to 1,000 bacteria per gram in the upper part of intestinal track, to 10^11^–10^12^ bacteria per gram in the colon, among them Firmicutes and Bacteroidetes present the main group (1). In contrast, the fetal intestine is sterile, the initial bacterial colonization at birth comes from the maternal microbiota, and the intestinal microbiota of an adult is composed of ~1 kg of bacteria, viruses, protozoans, fungi and archaea (3).

Intestinal microbiota is considered to be associated with the neuro–endocrine–immune pathways, generating the concept of the gut–brain axis. The first evidence of the gut–brain axis came from a work of an army surgeon through monitoring gastric juices secreted by intragastric fistula he found that intestinal function was related with mood (4). About 60% of anxiety and depression patients are described to have intestinal function disturbance, such as in irritable bowel syndrome (IBS). Recently, IBS has also been related to changes in intestinal microbiota, including reduced microbiota species and genus potent instability. Meanwhile, many animal studies suggest that intestinal microbiota disturbances could induce an increased visceral pain response and changes of brain chemistry and behavior (5). One study proposed that acute mania patients had more antibiotics prescriptions (6). For female patients these prescriptions were related to urinary-tract infections, while for male patients prescriptions were for respiratory and skin surface infections, the study showed that the increased antibiotics prescriptions was related with mania severity (6). The authors proposed three possible mechanisms: (i) potent infection activated the immune system and then induced mania; (ii) high bacterial infections in mania patients reflected the low response state of the immune system; and (iii) the usage of antibiotics could have changed the microbiota, which itself could increase the risk of mood state change (6). Intestinal microbiota can also produce some metabolic substances such as bile acids, choline and short-chain fatty acids, which can aid the hosts metabolism. Complex carbohydrates such as dietary fiber can be absorbed and fermented by intestinal microbiota into short-chain fatty acids such as butyrate, acetate, propionate, these all have neuroactive properties and can enter into blood and play a role in brain through two kinds of dispersed 7-transmembrane G protein coupled receptor such as free fatty acid receptor 2 (FFA2), FFA3 (7). Thus, intestinal microbiota plays an important role in mood disorders via the gut-brain axis, this review discusses elements of the gut-brain axis and discusses its relationship with mood disorders.

## Gut–brain axis and neuroendocrine

### Hypothalamic–pituitary–adrenal (HPA) axis

Acute stress is related with cortisone release through the HPA axis, a biological response to a sudden stressor, while chronic stress is related to an imbalance of the HPA axis. A study suggests that the acute stress is related with a potent adaptive immune response (inhibition of the intracellular pathogen immune, and preservation of the extracellular pathogen immune system), and chronic stress is the maladjustment of these two kinds of immune response (8). Meanwhile these two kinds of stress have different effects on intestinal microbiota. The effect of acute stress is limited due to microbiota's long time relative stable state, but chronic stress can disturb this kind of balance (8).

Stress can lead to changes in the microbiota and consequently on mood through the HPA axis. Corticotrophin-releasing factor (CRF) and CRF receptors (CRF 1, CRF 2) can play important roles in changes in intestinal permeability induced by stress. For acute stress, para-colonic permeability increases and is related with the development of visceral hypersensitivity. Early life stress can increase the cortisone level in plasma and its permeability, and promote bacteria to move to the liver and spleen (9). O'Mahony et al. found that microbiota in the feces of maternal-separate mice changed, in addition cortisone level increased, proinflammatory cytokine tumor necrosis factor alpha (TNF-α), Interferon-γ (IFN-γ) increased and Interluekin-6(IL-6) also had an increasing trend (10). The proinflammatory cytokine, IL-6 is an activator of the HPA axis; consistent activation of this axis can lead to a downregulation of glucocorticoid receptor, which is related with the feedback mechanism of HPA axis, and further leads to HPA axis over-activation making it harder to inhibit inflammatory reaction, this may explain the increased cortisone and proinflammatory cytokine (10). Park et al. found that depression and anxiety model rats show changes in colonic movement and intestinal microbiota, meanwhile motor neuropeptide, intestinal hormone, serotonin (5-HT) also increased which could be a presumptive factor of changes in intestinal movement. In these rats, corticotrophin-releasing hormone (CRH) expression increased in the paraventricular nucleus area of hypothalamus. In contrast, administering CRH to normal rats showed behavioral and intestinal changes similar with the changes induced by stress, strongly suggesting that the depression was the result of changes in intestinal microbiota through HPA axis (5).

Microbiota can relieve depression and anxiety. Crumeyrollearias et al. using gut microbiota-free (GF) rats and special pathogen-free (SPF) rats found that the lack of intestinal microbiota increased anxiety-like behavior when facing novelty challenge or being exposed to an open strong light environment (open field test) and these increasing behaviors were accompanied with aggravated HPA axis response. The GF rats presented with increased cortisone concentration in serum after acute open field stress test compared with SPF rat. These findings coincide with an increase in the growth-regulating factor (GRF) gene expression in the hypothalamus and a decrease in the hippocampus. (11). Bravo et al. found that *Lactobacillus rhamnosus* could reduce the increase of cortisone induced by stress. Another study found a similar result that the use of antidepressants could prevent the increase of cortisone induced by the enforced swimming stress test (12). There is also study reporting that *Lactobacillus* and *Bifidobacterium* could reverse the regulation of HPA axis (12).

All of these studies indicate that stress activates the HPA axis and increases cortisone level leading to increases in anxiety levels, intestinal movement and intestinal microbiota changes. However, intestinal microbiota can also inhibit the increase of cortisone through HPA axis to relieve anxiety and depression. Therefore, the HPA axis plays a key role in the bi-directional regulation of the gut-brain axis.

## Gut–brain axis and neurobiochemistry

### Neurotransmitter

Microbiota can secrete many kinds of neurotransmitters, for example *Lactobacillus* subspecies can secrete acetylcholine (regulating memory, attention, learning and mood), *Candida, Streptococcus, Escherichia coli* and *Enterococcus* can secrete 5-HT, and Bacilli and *Serratia* can secrete dopamine (13). Some intestinal microbiota such as *Lactobacillus acidophilus, Bifidobacterium infantis, Bifidobacterium, Candida, Streptococcus* have proven mental illness therapeutic effects through secreting neurotransmitters (gamma-aminobutyric acid [GABA], 5-HT, glycine, catecholamine) or regulating endocannabinoid expression. Neuroactive molecules secreted by intestinal microbiota have the ability of regulating nerve signals and can affect neuropsychiatric parameters such as sleep, appetite, mood and cognition (14).

Some neurotransmitter changes in stress state may not be caused by stress itself, but by the intestinal microbiota. Crumeyrollearias et al. found that in GF rats homovanillic acid (HVA) concentrations decreased in the anterior cortex, hippocampus, and striatum and lead to low HVA/dopamine, seen as the reduction of dopaminergic conversion rate (11). This kind of change was unrelated with open field stress since the effects of the open field stress showed concentrations of norepinephrine, 5-HT, and its metabolite 5-hydroxyindole acetic acid (5-HIAA) in the anterior cortex and the hippocampus were the same in both GF or SPF rats. The low dopaminergic conversion rate in GF rat might be due to a reduction in DA degradation enzyme numbers or activities such as catechol methoxy methyltransferase and monoamine oxidase (11). Depression is associated with a downregulation of tryptophan metabolism presented by the increased plasma ratio of kynurenine/tryptophan. Kelly et al. showed that GF rats who were administered microbiota from depressed patients also presented with an increase in HVA/dopamine ratio (15).

However, another study proposed a gender factor and the relationship between anxiety-like behavior and 5-HT. Clarke et al. found increased 5-HT and 5-HIAA in GF rats and this kind of change was only observed in male rats, but both genders presented with the same immune and neuroendocrine aspects (16). This kind of gender difference might be related to the effect of the animal estrus cycle hormone on the central nervous system 5-HT system, which could have a stronger effect than the transplanted microbiota. Meanwhile, the increase of tryptophan, a 5-HT precursor, prompted a possible humoral pathway of microbiota acting on the neural system, shown in a study that detected a tryptophan change after giving probiotics (16). Moreover, the 5-HT increase did not change after giving normal microbiota transplantation to GF rats post-weaning even though tryptophan had recovered, which indicated that the neurobiochemical effect due to a lack of microbiota in early life was hard to reverse (16). This period is associated with the development of the hippocampus and serotonergic system where the first neurons develop (16). They also found that male GF rats presented decreased anxiety-like behavior in light-dark box test, from this we can find that the reduced anxiety-like behavior seems not to correspond with the elevation of 5-HT activity and moreover, the reduced anxiety-like behavior could recover after giving normal microbiota transplantation to GF rats post-weaning while the increased 5-HT could not (16). Another study confirmed that Lactobacilli could reduce the anxiety-like and depression-like behavior, concluding that anxiety might not be caused by the disconnection of gut–brain axis and microbiota but the psychiatric development problem. The relationship between 5-HT and anxiety cannot be explained simply by anxiety or anti-anxiety alone (16). Although we use selective serotonin reuptake inhibitors (SSRIs) to treat anxiety, there is evidence that a diet lacking tryptophan or SSRIs had no effect on anxiety-like behavior caused by light-dark box test, which also indicates that the elevation of 5-HT in the hippocampus and anxiety-like behavior could be associated with a separation relationship (16).

### Brain-derived neurotrophic factor (BDNF)

Mood disorders have been linked to the activity and productive state of neurotrophic substances specifically BDNF, thus increasing BDNF levels can be an efficient intervention (17). A range of research address this issue, such as administering *Bifidobacterium* can increase BDNF in the hippocampus, administering antibiotics which increases the amount of *Lactobacillus* in the intestinal tract can increase BDNF in the brain, and administering prebiotics such as fructo-oligosaccharide and galacto-oligosaccharide can increase BDNF in the hippocampus and periphery (17). Clarke et al. found a reduction in BDNF mRNA expression in the hippocampus of GF male rats. BDNF is an important neurotrophic substance for the development and repair of the hippocampus, however, neither the normal group or GF group presented differences in the weight of the hippocampus regardless of gender, therefore they suggest the reduction of BDNF is a compensation for the elevation of 5-HT (16).

### Vagus nerve

The intestinal tract is controlled by intrinsic and extrinsic factors, the intrinsic factor is intestinal neural system also called the second brain which is composed of 200–600 million neurons (2). The intestinal neural system is also controlled by extrinsic factor including the vagal and sacral parasympathetic nerve fibers and visceral sympathetic nerve fiber (2). The vagus nerve is the primary connection between the brain and proximal intestinal tract, sacral parasympathetic nerve fiber connects the distal third colon (2). The humoral connection includes the HPA axis, which is responsible for regulating stress response and intestinal endocrine cells, secreting neuropeptides and intestinal peptide that act locally and through the vagus nerve and spinal cord afferent or blood brain barrier to act on the brain (2).

Bravo et al. showed that *L. rhamnus* regulated behavioral and physical reactions through the vagus nerve, by regulating GABAAα2, GABAAα1, GABAB1b receptors mRNA expression (all are anxiety behavior related receptors) (12). Previous studies have shown increased GABAAα2 mRNA levels in the amygdala of stressed animals. The amygdala can regulate fear and anxiety behavior, thus indicating that reduced GABA receptor subtypes induced by *L. rhamnus* has adaptive advantage in stress state, and the expression differences of GABAAα2 and GABAAα1 in rat hippocampus after administering *L. rhamnus* could also explain the low anxiety-like and depression-like behavior observed (12). Interestingly, in vagotomy rats this kind of neurochemical and behavioral changes were not detected, indicating that the vagus nerve is an important pathway for regulating the gut–brain axis and microbiota (12).

Pathological ecological imbalance can increase intestinal vagus nerve activity, inducing low grade intestinal inflammatory reaction leading to anxiety-like behavior in animals, but in vagotomy animals this kind of behavioral disturbance is not seen (18). Some studies showed that administering probiotics *Bifidobacterium longum* to rats that had intact vagus nerve could prevent the intestinal inflammatory reaction related with anxiety-like behavior, but to those conducting vagotomy after chronic low grade inflammatory reaction, antidepressant effects were not observed (18). Therefore, the relief of anxiety after administering probiotics was apparently not related with the relief of inflammatory reaction itself (18). In other words, the vagus nerve could be seen as the pathway of behavioral change, having anxiety (through intestinal inflammatory reaction) and anti-anxiety (through probiotics) effects (18).

## Gut–brain axis and the immune system

### Lipopolysaccharide

The imbalances in the intestinal tract and the changes in permeability lead to the production of a kind of proinflammatory endotoxin—lipopolysaccharide (LPS), which can enter the blood system. LPS is important for regulating the neural system, it increases the activity of the amygdala which is responsible for regulating emotion, affecting the physical activity of the brain and regulating the further production of neuropeptides (4). LPS when given to healthy people can produce inflammatory cytokine and increase norepinephrine in plasma, which has been linked to high depression mortality (4).

Some studies have shown that just a small amount of LPS could lead to symptoms of acute anxiety and depression, cognition deficit and an increase in visceral pain sensitivity (19). LPS is a component of the outer membrane of Gram-negative bacteria, there is about 1 g of LPS in the human intestinal tract, and in a healthy state there is only a small amount of LPS present in plasma due to the effective rejection of intestinal barrier (19). The inflammation induced by LPS can increase indoleamine 2,3 dioxygenase (IDO, a kind of tryptophan decomposing enzyme through kynurenine pathway) activity, which is correlates positively with symptoms of depression. Kynurenine itself can also lead to anxiety when acting on the periphery, but in general kynurenine produced by the intestinal microbiota or coming directly from food can be quickly absorbed and play a role in anti-anxiety (19). LPS can damage the blood–brain barrier and consequently allow a large amount of potent substances to cross, such as environmental toxins, pathogens and even itself (19). Evidence confirms that depression is accompanied with the activation of an inflammatory response, proinflammatory cytokines, and LPS can also lead to symptoms of depression (20). Maes et al. found increased Immunoglobulin M (IgM) and IgA in the serum of patients with major depression, which were associated with intestinal microbiota LPS. These increases corresponded with fatigue, autonomic nerve and gastrointestinal symptoms, as well as the subjective infectious “sickness” feeling. The increase of migration of Gram-negative bacteria triggers an intestinal mucosal dysfunction playing an important role in the inflammatory pathological changes of depression. The increased LPS migration could increase the immune response and further activate the inflammatory system inducing the reported “sickness” feeling (20).

### Proinflammatory cytokine

Stress can lead to changes in the intestinal barrier function, enabling different molecules to enter into the blood and immune system. Stress can produce a proinflammatory reaction and produce high levels of proinflammatory IL-1 and IL-6 (3). A study claim that depression is related with the increase of inflammatory bio-markers such as IL-6, TNF-α and C-reactive protein (21). Recently depression was redefined into a clinical expression of activated immune inflammatory, oxidative and nitrosative stress (IO&NS) pathway, including tryptophan metabolism (TRYCAT), autoimmune and gut–brain pathway (22). The IO&NS pathway is important in IBS, the high depression mortality rate in IBS patients and the high comorbidity of IBS in patients with depression indicates that depression may be able to regulate the pathological change of IBS. These two diseases have been associated with several changes in the IO&NS pathway, such as increased inflammatory cell level (such as IL-1, TNF-α, IL-6, T helper cell 1 (Th-1) and Th-17-like reaction, neopterin and soluble IL-2 receptor level, globin and C-reactive protein), reduced negative acute phase reaction and anti-inflammatory cytokines (IL-10, transforming growth factor-β (TGF-β), increased damage to lipid, protein and DNA, increased nitric oxide and inducible nitric oxide synthase products, reduced tryptophan concentration in plasma but increased TRYCAT, and increased autoimmune response and bacterial translocation (22). This kind of overlapping IO&NS pathway of depression and IBS can explain why an increase in comorbidity is reported (22).

Winther et al. showed that rats given a diet lacking in magnesium for 6 weeks presented fewer behavioral characteristics associated with depression in the forced swim test, their intestinal microbiota changed, and IL-6 decreased in the hippocampus. These results might be because the cells producing cytokine are in a low metabolism state due to lack of Mg. A previous study has indicated that IL-6 in hippocampus could induce depression-like behavior in the forced swim test. Interestingly, the microbiota constitution of these rats lacking magnesium was highly related with IL-6 levels in the hippocampus, which indicates that diet can induce changes in the microbiota constitution and change behavior through immune regulation (23). Some proposed that besides acting on central monoamine substances, antidepressants can also inhibit the inflammatory reaction and produce potent immune regulation of the cytokine IL-10. Interestingly, probiotics microbiota regulate the immune system through acting on T regulating cells and the secretion of IL-10 (24). Some previous studies proposed that administering commensal bacteria to GF rats can lead to local dendritic cell uptake and transform into phenotype that promote T regulating cells production and IL-10 synthesis, administering *Lactobacillus GG* was also shown to be able to increase IL-10 in some patients (24).

### Other immune inflammatory factors

Wong et al. found that a lack of caspase-1 could decrease depression-like and anxiety-like behavior, meanwhile prevent deterioration of depression-like behavior after a stressful event. They also found that administering an antagonist of caspase-1 in a chronic stress situation had similar ways in changing intestinal microbiota. When using minocycline (caspase-1 antagonist) to treat stressed rats, they observed an increased abundance of *Akkermansia* spp. and *Blautia* spp. (associated with a weakening inflammatory reaction and rebalancing of intestinal microbiota, respectively) and increased *Lachnospira* (associated with the changes caused by a lack of caspase-1) (25). All of these studies indicate that inhibiting caspase-1 could produce a protective function to regulate stress levels and change intestinal microbiota. Intestinal microbiota can change brain function and affect depression-like and anxiety-like behavior through an inflammatory signal pathway (25). Martín-Hernández et al. reported that rats had higher intestinal permeability and bacterial translocation after chronic stress, the expression of the activated form of mitogen activated protein kinase (MAPK) p38 increased but the expression of anti-transcriptional factor Nrf2 (antioxidant) decreased (25). Due to the fact that antibiotics prevent bacterial translocation through MAPK and Nrf2 pathways, these results indicate that the translocated bacterial participated in these effects, which meant the translocated bacteria could be related with the pathology of MAPK p38 pathway participating in depression, deteriorating neural inflammation and oxidation/nitriding destruction (26).

## The constitution of intestinal microbiota and mood disorders

The constitution of intestinal microbiota in humans is determined by the type of delivery at birth, gene tendency, age, nutrition, physical activity, environmental factors, stress, infection, other disease and the usage of antibiotics. Genes of the brain and intestinal tract are quite similar, especially related to the formation of the neuronal synapse, thus some gene mutations can lead to abnormalities in both the brain and gut (21).

Stress factor or mood disorders can make changes in the constitution of intestinal microbiota. In a prenatal stress rat model, maternal stress could reduce the abundance of *Lactobacillus* in the vagina and reduce its transmission to the offspring, further leading to changes in metabolism linked to energy balance and damage to amino acid configuration in brain development (1). In another study, the children from mothers with high reported stress and high saliva cortisol concentrations presented prominently with a high level of *Bacillus proteus* (control germs) abundance and relatively low levels of *Lactobacillus* and *Bifidobacteria* abundance (1). It is unknown if this kind of influence is due to microbiota transmission from mother or the specific effect of cortisone on the intestinal tract development (1). Whatever the mechanism, the children with microbiota changes presented higher level of intestinal symptoms and allergic reaction, raising the attention on the functional change caused by abnormal colonization pattern (1). Studies claim that stress hormone promotes the growth of non-pathogenic bacteria *Escherichia coli* and pathogenic bacteria *E. coli* 0157:H7 through the interaction with the host's catecholamine adrenaline and norepinephrine. Further studies claim that the stress before birth could change the microbiota constitution in rhesus monkeys: inducing the total number of *Bifidobacterium* and *Lactobacillus* (27). Murakami et al. studied the influence of maternal separation (MS) and stress caused by CRH to intestinal microbiota and colorectal movement. The experimental group were MS group, control group were normal rats, CRH or saline were administered after 8 weeks, they found that both kinds of stress could change the microbiota constitution in feces, and among those given CRH, MS rats presented more colorectal movements (28). The human studies confirmed the same results. Kelly et al. proposed that depression was related with the constitution, amount and species of intestinal microbiota, and they could distinguish between the patients with depression and healthy people through the species of microbiota. Both the depressed patients and GF rats who were transplanted microbiota from depressed patients presented reduced abundance degree and alpha diversity (15). Aizawa et al. found that the amount of *Bifidobacterium* and *Lactobacillus* in the feces of patients with major depression was less than that of normal people, and among the patients with major depralphaession, those who drank yogurt products had more *Bifidobacterium* in their feces (29). Evans et al. analyzed the feces microbiota constitution of 115 bipolar disorder patients and 64 normal people and found that bipolar disorder patients had fewer *Faecalibacterium*, and the proportion of *Faecalibacterium* was related with better self-reported health outcomes based on SF-13 (simple SF-12 health survey questionnaire), PHQ-9 (Patient health questionnaire-9), PSQI (Pittsburgh sleep quality index), GAD7 (Anxiety screening questionnaire), and ASRM (Altman manic scale) (30).

Different microbiota constitution can produce symptoms of mood disorders. The total number of bacteria predicts insomnia problems and high depression score in QIDS-SR (Quick Inventory of Depressive Symptomatology), reduced *Enterobacteriaceae* predicts the high anxiety score in GAD7, and in depressed patients, *Lactobacillus*/*Enterococcus* is positively related with psychomotor agitation items in QIDS-SR and GAD-7 (31). Zheng et al. showed a lack of microbiota induced depression-like behavior, the microbiota constitutions of major depression disorder (MDD) patients and healthy people were different and this kind of difference had transmissibility, GF rats who were transplanted with “depression microbiota” presented more depression-like behavior compared with those who are transplanted with “normal microbiota” (1). Kelly et al. came to a similar conclusion: GF rats who were transplanted with microbiota from depressed patients presented with the development of depression-like features such as anhedonia and anxiety-like behavior and produced similar physiological features as depressed patients (15). GF rats transplanted with microbiota from depressed patients mainly presented with disturbances in the microbiota gene and host metabolism, including the metabolism of carbohydrates and amino acids, which indicated that intestinal microbiota played a role in depression through their effects on the host metabolism (32). Some healthy human studies proposed no relationship, Kleiman et al. used 91 healthy female adults and found no physiological and psychological relationship between the constitution, species of intestinal microbiota and psychological measures (such as depression, anxiety, eating behavior, behavior, stress and personality) (33).

All of these studies indicate that the constitution of intestinal microbiota may present a bi-directional relationship with mood disorders.

## Prebiotics and mood disorders

Some studies look at the effects of prebiotics and mood disorders, such as fructo-oligosaccharide (FOS) and galacto-oligosaccharide (GOS), which are the nutrition substances of *Bifidobacterium* and *Lactobacillus* (34). Administering prebiotics to rats can increase the expression of BDNF in the hippocampus and the mRNA expression of BDNF in the dentate gyrus, as well as the increasing the expression of a N-methyl-D-aspartic acid (NMDA) receptor subtype, all of which can influence synaptic plasticity and memory function (34). However, in human studies, administering FOS, GOS or a placebo for control showed that groups given B-GOS presented with a reduced awakening cortisone reaction (the increasing awakening cortisone is the biomarker of emotion disturbance such as depression), reduced vigilance and reduced attention to negative emotion, indicating an anti-depressant and anti-anxiety function (34). Evidence suggests that prebiotics saliva lactose could reduce colonic mucosa-related microbial community structure change induced by stress, FOS could increase BDNF expression and NMDA receptor signal, administering GOS to a human group inhibited the neuroendocrine stress response, the increase of positive and negative process of attention vigilance and early anti-anxiety pattern (35). Burokas et al. administered rats FOS, GOS and their combination and found that prebiotics reduced anxiety-like behavior in an open-field and elevated maze test, and depression-like behavior in tail suspension and forced swim test. Meanwhile, they presented with lower cortisone and L-tryptophan level in plasma caused by stress. These effects were the most prominent in the combination group, which might be due to a wider range of bacterial stimuli. Besides the blunt aggressive behavior and more pro-social methods, no other cognitive, pain or social changes were observed. The behavioral changes in rats corresponded with a monoamine level change, the combination group presented with high levels of BDNF in the hippocampus and the mRNA expression increase of GABAb receptor subtype in hippocampus. Another change that could explain behavioral change was the elevation of 5-HT in the prefrontal cortex and a trend of 5-HT elevation in the frontal cortex (35). These studies confirm that prebiotics can relieve mood disorders and also indicates that microbiota can act on mood disorders.

## Probiotics and mood disorders

The relative mechanism of probiotics are: regulating the capacity of intestinal microbiota, preserving the integrity of the intestinal barrier, preventing bacteria translocation and regulating local inflammatory reaction through intestinal related immune system (17).

Probiotics can regulate the intestinal microbiota constitution. The case report of Schnorr and Bachner showed that giving food containing probiotics to a patient with anxiety resulted in an increase in total sleep time in the third week, which was helpful to relieve chronic stress, a decreased Beck Anxiety Inventory score in the second week, and a constitutional change in microbiota after 2–3 weeks: *Lactobacillus* and *Bacteroides* increased, *Clostridium* family (*spirobacteria, Blautia*) decreased, *Actinomycetes* decreased (Collins bacteria mainly decreased) (36). Both in the aspects of phylogenetic classification or the classification of the same species, the species of microbiota increased (36). All of these results indicate that giving probiotics could improve and redistribute species of microbiota to relieve anxiety behavior (36).

Probiotics could restore the integrity of colonic tight junction in stressed rats. A previous study claimed that *Lactobacillus farciminis* could not only inhibit permeability, the HPA axis activity, endotoxemia, neuroinflammatory changes induced by stress, but also generate beneficial effect on mucosal barrier (9).

Probiotics can reduce proinflammatory cytokines and oxidative stress in human, both are related with mood disorders. Romijn et al. found that the psychological results of those with high vitamin D from the initial time of study presented with improvements after giving probiotics (37). Since vitamin D can regulate the immune system, they thought the vitamin D state of the host might be associated with the relationship between intestinal microbiota and immune system (37).

Probiotics can relieve depression and anxiety symptoms. Messaoudi et al. found that rats given probiotics performed better than those given placebo and similar to diazepam as the standard reference substance when conducting an anxiety degree test (38). And among a sample of healthy people, those given probiotics presented with lower total severity scores (lower somatic, depressed, and angry enemies scores) in HSCL-90 (Hopkins symptom checklist) compared with those given placebo, and a decreased HADS (Hospital Anxiety and depression scale) scores. As for CCL (Coping Checklist), which aimed to assess the solving strategy when facing life stress, those given probiotics showed a lower self-accusation scores, and the control group showed higher positive re-evaluation scores (38). Furthermore, compared with a control group, the probiotics group focused more on solving problem and their cortisone levels also decreased (38).

However, in human samples there are some different results. Cepeda et al. used transection analysis based on big data in the USA to assess whether probiotics had an effect on depression. They found that those consuming probiotics had lower depression mortality, but when excluding the sample characteristic factor, this kind of difference became less prominent (39). Therefore, they thought the reason of this result was the features of those consuming probiotics people themselves, such as a healthier lifestyle, which in itself has a lower risk of depression, thus concluding depression had no relationship with probiotics (39). Romijn et al. used 79 participants who were assessed with scales and had at least moderate scores, and gave them combined probiotics (*Lactobacillus helveticus* and *B. longum*) or placebo for 8 weeks. They found that these two groups presented no differences in psychological results (37). But previous studies actually proved these two kinds of probiotics had a positive effect on the emotional behavior of animals and the psychological result of human samples. They suggest that the reason for their result was that their intervention time or the amount of samples were sufficient, when using probiotics as a single treatment, it might need more than 8 years to have an effect on mood (37). Meanwhile the severity of samples and their course of disease might also be a factor since 70% of the chosen samples had persistent low mood for more than 2 years; probiotics may be efficient just to those with short course or with not so serious low mood (37). Otherwise considering most of the participants had received antidepressant treatment before, they may present with treatment resistance (37).

## Antibiotics and mood disorders

Some studies presented that the rats that were given neomycin, bacitracin and antifungal drugs natamycin to induce dysbacteria showed lower anxiety-like behavior in a step-down test and light-dark box test. In addition, their BDNF expression in the amygdala also changed, but after drug withdrawal, these behavioral changes restored (40). Similarly, using citrobacter to create microbial infection presented increased anxiety-like behavior in the Hole-board open field apparatus and memory dysfunction induced by stress, but given to GF rats, once infected, they presented with memory dysfunction whether giving stress or not (40). Desbonnet et al. studied the rats who were given antibiotics from weaning and found their intestinal microbiota changed: they had relatively less *Firmicutes* and *Bacteroidetes*, relatively more *Cyanobacteria* and *Proteobacteria*, and reduced anxiety in light-dark box test, cognition deficient, changed tryptophan metabolic pathway, reduced expression of BDNF, oxytocin and antidiuretic hormone in the brain of adult rats. This kind of effect on anxiety was only associated with the antimicrobial function of antibiotics (41).

## Conclusion

We can conclude that intestinal microbiota has a bi-directional effect on mood disorders through the mechanisms of the neurobiochemical, neuroendocrine, and neuroimmune systems of the gut-brain axis. Different intestinal microbiota constitutions can change the symptoms of mood disorders, meanwhile mood disorder itself can change the constitution of microbiota. Through analyzing these studies on prebiotics, probiotics and antibiotics, we can not only further show that there is a relationship between microbiota and mood disorders, but also find that administering prebiotics, probiotics and suitable antibiotics can relieve depression and anxiety symptoms, providing alternative therapeutic strategies to treat mood disorders. However at present stage, most of the relative studies are based on animal models, only a few of them are human studies. Among these human studies, prebiotics have been proved of having relationship with mood disorders, but the relationship between the constitution of intestinal microbiota, probiotics and mood disorders has not reached a consensus. And there is no any human studies on antibiotics. Therefore, in the future more human studies need to be done.

## Author contributions

GZ designed and corrected the paper. LL wrote the paper.

### Conflict of interest statement

The authors declare that the research was conducted in the absence of any commercial or financial relationships that could be construed as a potential conflict of interest.
